# Atypical McMurry Cross-Coupling Reactions Leading to a New Series of Potent Antiproliferative Compounds Bearing the Key [Ferrocenyl-Ene-Phenol] Motif

**DOI:** 10.3390/molecules190710350

**Published:** 2014-07-17

**Authors:** Pascal Pigeon, Meral Görmen, Konrad Kowalski, Helge Müller-Bunz, Michael J. McGlinchey, Siden Top, Gérard Jaouen

**Affiliations:** 1 Chimie ParisTech, 11 rue Pierre et Marie Curie, Paris F75231 Paris cedex 05, France; E-Mails: pascal.pigeon@chimie-paristech.fr (P.P.); meralgormen@gmail.com (M.G.); 2 Sorbonne Universités, UPMC univ Paris 6, Institut Parisien de Chimie Moléculaire (IPCM) - UMR 8232, 4 place Jussieu, 75252 Paris Cedex 05, France; 3 Faculty of Chemistry, Department of Organic Chemistry, University of Łódź, Tamka 12, 91-403 Łódź, Poland; E-Mail: konkow@chemia.uni.lodz.pl; 4 UCD School of Chemistry and Chemical Biology, University College Dublin, Belfield, Dublin 4 Ireland ; E-Mails: helge.muellerbunz@ucd.ie (H.M.-B.); michael.mcglinchey@ucd.ie (M.J.M.)

**Keywords:** DES, ferrocene, pinacol rearrangement, McMurry reaction, antiproliferative activity, MDA-MB-231

## Abstract

In the course of the preparation of a series of ferrocenyl derivatives of diethylstilbestrol (DES), in which one of the 4-hydroxyphenyl moieties was replaced by a ferrocenyl group, the McMurry reaction of chloropropionylferrocene with a number of mono-aryl ketones unexpectedly yielded the hydroxylated ferrocenyl DES derivatives, **5a**–**c**, in poor yields (10%–16%). These compounds showed high activity on the hormone-independent breast cancer cell line MDA-MB-231 with IC_50_ values ranging from 0.14 to 0.36 µM. Surprisingly, non-hydroxylated ferrocenyl DES, **4**, showed only an IC_50_ value of 1.14 µM, illustrating the importance of the hydroxyethyl function in this promising new series. For comparison, McMurry reactions of the shorter chain analogue chloroacetylferrocene were carried out to see the difference in behaviour with mono-aryl ketones *versus* a diaryl ketone. The effect of changing the length of the alkyl chain adjacent to the phenolic substituent of the hydroxylated ferrocenyl DES was studied, a mechanistic rationale to account for the unexpected products is proposed, and the antiproliferative activities of all of these compounds on MDA-MB-231 cells lines were measured and compared. X-ray crystal structures of cross-coupled products and of pinacol-pinacolone rearrangements are reported.

## 1. Introduction

**Scheme 1:**
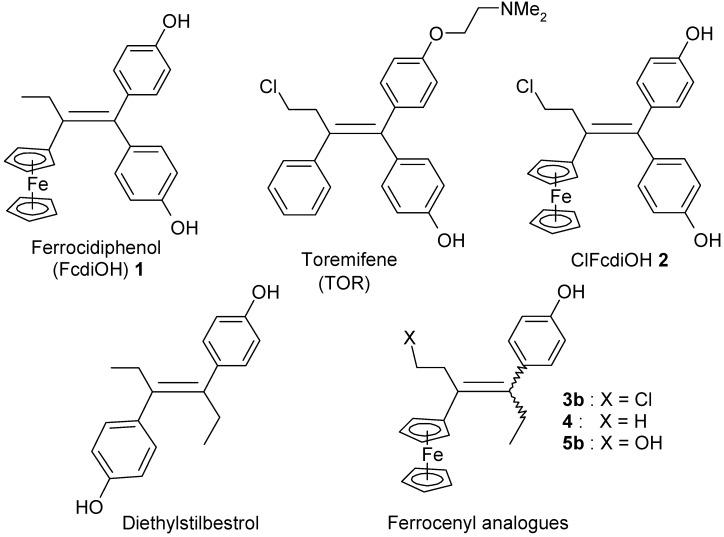
Toremifene, ferrocidiphenol, ClFcdiOH, DES and the new Fc-DES series.

Bioorganometallic chemistry, otherwise known as organometallic chemical biology, a field that encompasses organometallics in biology and medicine, is one of the fastest growing fields. Indeed, many organo-transition metal complexes have attracted great interest in a wide range of medicinal areas [1,2,3,4,5,6,7]. As part of our programme to study ferrocene derivatives with anticancer properties, we have previously reported that ferrocidiphenol (4-HO-C_6_H_4_)_2_C=C(Et)Fc, **1**, [8,9] shows an effective cytostatic effect (IC_50_ = 0.7 µM on hormone-dependent MCF-7 and IC_50_ = 0.6 µM on hormone-independent MDA-MB-231 breast cancer cell lines) [10]. We chose to prepare this antiproliferative species, modelled on tamoxifen (TAM), but lacking the aminoalkyl chain which is irrelevant for the MDA-MB-231 cell line devoid of the estrogen α receptor (ERα). Toremifene (TOR, Scheme 1), a chlorinated derivative of tamoxifen, is an effective and well-tolerated agent for the therapy of postmenopausal women with hormonal positive receptor advanced breast cancer [11,12,13,14]. Its citrate form is approved by the FDA and licensed in the United States under the brand name Fareston^®^. TOR is considered as an alternative to TAM as a first line therapy for ER+ advanced breast cancer patients, and also as an adjuvant treatment [15]. It decreases the incidence of prostate cancer in men with high grade prostatic intraepithelial neoplasia [16,17]. It also shows a benefit by increasing hip and spine bone mineral density in men receiving androgen deprivation therapy for prostate cancer [18]. By analogy to the structure of TOR, in which TAM has been modified by incorporation of a chloroethyl substituent, we synthesized **2**, the chloroethyl derivative of ferrocidiphenol, **1**, and found it to have low antiproliferative activity against MCF-7 and moderate activity on MDA-MB-231 (IC_50_ = 1 µM). This led us to postulate that a hydrophobic substituent on the ethyl group diminishes the antiproliferative efficiency. We have also previously suggested that, to be active, the redox-active ferrocifen complexes need to possess the key [ferrocenyl-ene-phenol] motif, to allow *in vivo* formation of an electrophilic quinone methide that we supposed to be the active metabolite [19].

Accordingly, we envisioned the synthesis of a series of new compounds having the minimum structural requirement for efficiency, *i.e.*, bearing only one hydroxyphenyl group. We chose also to replace the second phenolic group in **1** and **2** by an ethyl group, thus leading to a series of ferrocenyl complexes analogous to diethylstilbestrol (DES). Diethylstilbestrol, [(*E*)-3,4-[bis-(4'-hydroxyphenyl)-3-hexene], acts as a powerful estrogen via ERα and ERβ [20]. This compound was prescribed for the prevention of pregnancy complications, but was subsequently prohibited due to many adverse effects, including carcinogenicity and teratogenicity [21], increased incidence of reproductive and genital abnormalities [22]. We have previously reported the synthesis of organochromium complexes of hexestrol, a hydrogenated version of DES [23,24], and ferrocenyl derivatives of estradiol and of hexestrol are also known [25]. In this new series, our goal was to compare the effect of chloroethyl (as in compound **3b**) and ethyl (as in compound **4**) groups on the antiproliferative activity of these compounds, as had been done previously for molecules **1** and **2** [26].

Somewhat surprisingly however, attempts to prepare the aforementioned chlorinated compounds, via McMurry coupling of chloropropionylferrocene and 4-hydroxyphenyl-alkyl ketones of varying alkyl chain length, led instead to hydroxyethyl derivatives (**5a**–**c**) resulting from the replacement of chlorine atom by a hydroxyl group; in addition, compounds resulting from pinacol-to-pinacolone rearrangement were also isolated. We here report the results of these atypical McMurry coupling reactions, and make comparison with the more usual reactions seen when using 4,4'-dihydroxybenzophenone as the purely organic ketone counterpart.

## 2. Results and Discussion

### 2.1. Synthetic Aspects

**Scheme 2:**
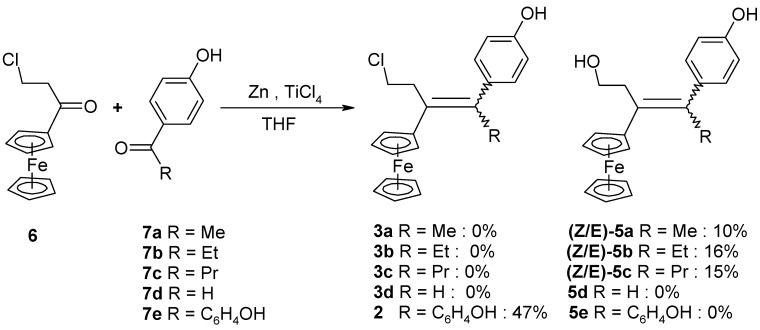
McMurry coupling reactions of chloropropionylferrocene, **6**. Two isomers, *Z* and *E*, were formed, and the designation *E* refers to the isomer whereby the ferrocenyl group is trans relative to the phenol.

Following the synthetic procedure successfully used for **2**, the chloroethyl derivative of ferrocidiphenol, **1** [26], we first performed a McMurry coupling between chloropropionylferrocene, **6** [27], and 4-hydroxypropiophenone, **7b** (Scheme 2). Surprisingly, only the hydroxyl compound **5b** was isolated, albeit in low yield (16%). The crude oil showed a mixture of inseparable compounds, **5b** being the only compound isolated by chromatographic techniques in pure form. This was attributable to its higher polarity induced by the hydroxyl group, as compared to the other by-products. Moreover, the expected chlorinated compound **3b** could not be detected by mass spectrometry in the reaction mixture. Again, when 4-hydroxypropiophenone, **7b**, was replaced by 4-hydroxyacetophenone, **7a**, or 4-hydroxybutyrophenone, **7c**, in the reaction with chloropropionylferrocene, **6**, only the hydroxyl compounds **5a** and **5c** were isolated, in yields of 10% and 15%, respectively. This contrasts with the previously observed reaction of 4,4'-dihydroxybenzophenone, **7e**, with **6** that furnished **2** (in 47% yield) [26] with no formation of hydroxylated compound **5e**.

These observations are apparently specific to the McMurry reaction between **6** and mono-aryl ketones, since the exchange of chlorine to hydroxyl was found only in these cases. The difference is almost certainly not a steric effect, although electronic effects such as π-stacking between a hydroxyphenyl group and the ferrocenyl moiety during the formation of the intermediate pinacolate [28] may play a role. The lower yields of cross-coupled product when using mono-aryl ketones **7a**–**c** with **6**, rather than the diaryl ketone **7e**, may be a result of increased homocoupling and also of pinacol-to-pinacolone rearrangement of intermediate titanium pinacolates. It is known that in McMurry reactions with two mono-aryl ketones there is less selectivity for cross-coupling than when using species as different as a mono-aryl and a diaryl-ketone. In our previous studies involving McMurry reactions between a mono-aryl and a diaryl ketone, the cross-coupling yields were generally quite high, homo-coupling was low, and pinacol rearrangement products were barely detectable. However, in the reactions of diaryl ketones and [3] ferrocenophanone [29], or of 4-hydroxybenzoyl-ferrocene and 4-hydroxypropio-phenone **7b** [30], rearrangement to pinacolones occurred. Replacement of the ketone by an aldehyde, as in the reaction of 4-hydroxybenzaldehyde, **7d**, with **6**, led to an inseparable mixture of unknown products, and neither the chlorinated compound **3d** nor the alcohol **5d** was detected by mass spectrometry.

Alcohols **5a**–**c** were obtained as mixtures of *Z*/*E* isomers. Even though the two forms could be separated by semi-preparative HPLC, the isomerization in solution did not allow the isolation of pure isomers except in the case of **5b** for which the major isomer could be isolated from crystallization and identified as a *Z* isomer by 2D NMR. However, this isomer slowly isomerized in acetone to give an 86:14 *Z/E* mixture. In **5a** and **5c**, the observed *Z/E* ratios in acetone were 73/27 and 62/38, respectively. All reactions produced mixtures, and only the hydroxyl compounds could be isolated in pure form, albeit in relatively low yields.

**Figure 1:**
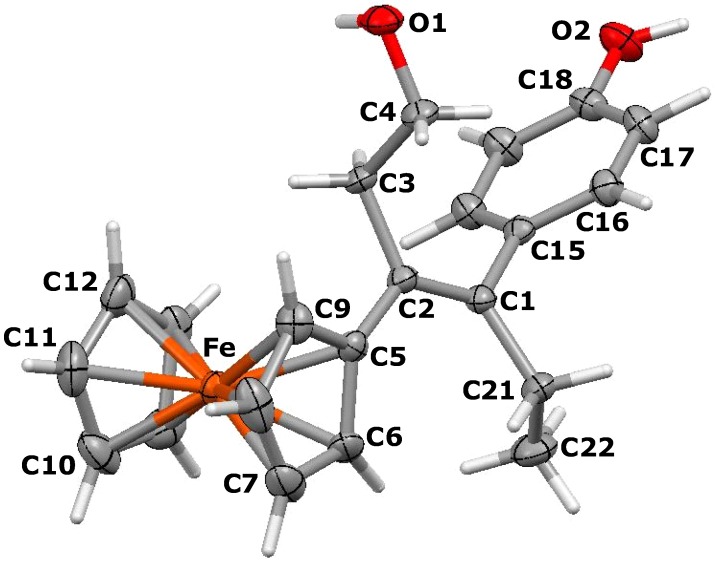
Molecular structure of *E*-3-ferrocenyl-4-(4-hydroxyphenyl)-1-hydroxy-hex-3-ene, **5b**. Thermal ellipsoids are shown at 25%. Selected bond distances (Å) for **5b**: C(1)-C(2) 1.339(5), C(1)-C(15) 1.506(5), C(1)-C(21) 1.512(5), C(21)-C(22) 1.526(6), C(2)-C(5) 1.485(5), C(2)-C(3) 1.531(5), C(3)-C(4) 1.515(5), C(4)-O(1) 1.433(4), C(18)-O(2) 1.377(5), Fe-centroid(C_5_H_4_) 1.634, Fe-centroid(C_5_H_5_) 1.640.

Suitable crystals of the *E* isomer of 3-ferrocenyl-4-(4-hydroxyphenyl)-1-hydroxy-hex-3-ene, **5b**, could be isolated for an X-ray crystallographic study. The molecular structure appears as Figure 1, and reveals that the ferrocenyl moiety is twisted out of the plane of the double bond though 30.5°, whereas the plane of the phenol ring is rotated through 68.6°, making a dihedral angle between the C_5_H_4_ and aryl rings of 81.8°. The ethyl and hydroxyethyl substituents lie on opposite sides of the central plane and make a dihedral angle of 151.6°. Clearly, these orientations minimise steric interactions between the bulky substituents. The cyclopentadienyl rings in **5b** are parallel and are separated by 3.274 Å.

**Scheme 3:**
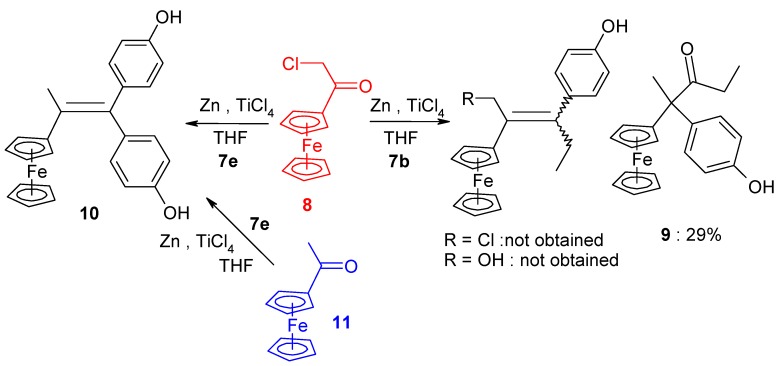
McMurry reaction between **8** (or **11**) and **7b** or **7e**.

Interestingly, when a shorter chain compound such as chloroacetylferrocene **8** [31] was allowed to react with the mono-aryl ketone **7b**, a different process occurred. As shown in Scheme 3, neither of the anticipated ethylenic compounds (chlorinated or hydroxylated) was formed, instead only the dehalogenated pinacolone **9** was isolated, in 29% yield. This compound corresponds to the formation of the more stable cation, situated α to the ferrocenyl moiety, with subsequent migration of the phenolic group onto the carbon bearing the positive charge. In principle, two pinacol-to-pinacolone rearrangement products are possible, but the migratory aptitude of the phenolic group is markedly higher than that of the alkyl substituent because of the greater ability of the former to delocalize positive charge in the transition state [32].

**Figure 2:**
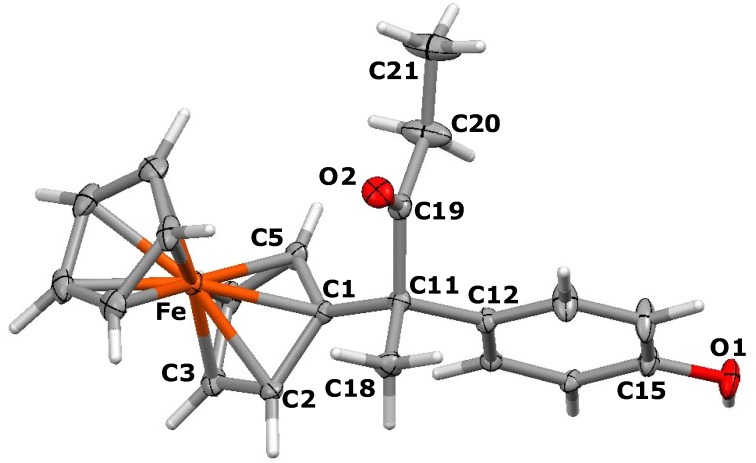
Molecular structure of **9**; thermal ellipsoids are shown at 50% probability. Selected distances (Å): C(11)-C(1) 1.544(3), C(11)-C(18) 1.564(3), C(11)-C(12) 1.546(3), C(11)-C(19) 1.550(3), C(19)-C(20) 1.505(4), C(19)-O(2) 1.223(3), C(15)-O(1) 1.376(3), Fe-centroid(C_5_H_4_) 1.664, Fe-centroid(C_5_H_5_) 1.667.

The identity of the pinacolone rearrangement product, **9**, was determined not only spectro-scopically, but also by X-ray crystallography (Figure 2), which established unequivocally that the phenolic group had migrated onto the carbon atom adjacent to the ferrocenyl substituent. The geometry at the central carbon, C(2), is almost perfectly tetrahedral with angles ranging only from 109.1° to 110.2°. The cyclopentadienyl rings in the ferrocenyl unit are parallel, and are separated by 3.331 Å; they make a dihedral angle of 85° with the plane of the phenol.

**Scheme 4:**
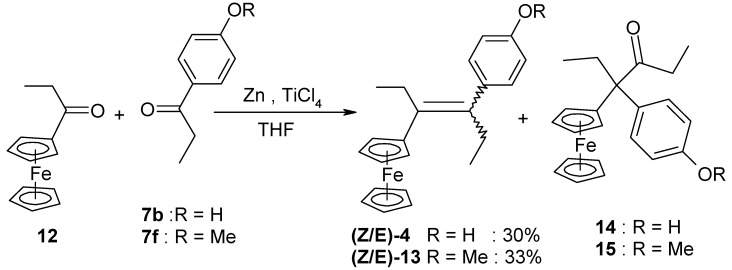
Synthesis of **4** and **13**.

**Figure 3:**
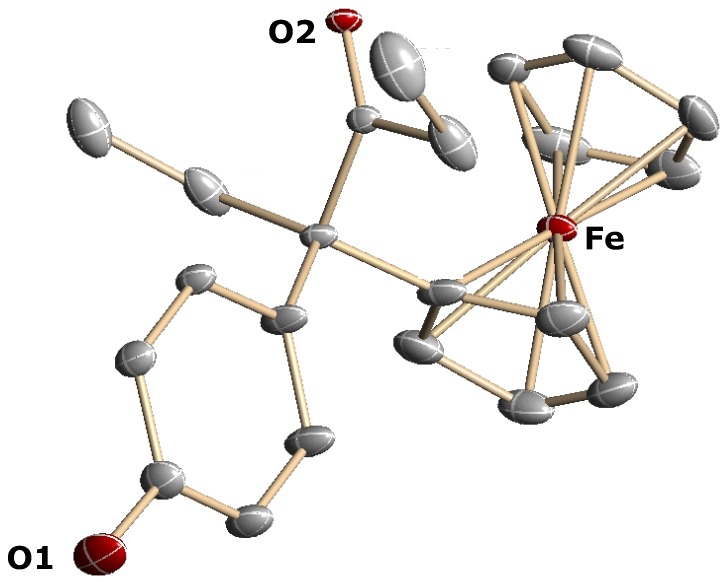
Molecular structure of 3-ferrocenyl-3-(4-hydroxyphenyl)-hexan-4-one, **14**.

In contrast, the reaction of chloroacetylferrocene, **8**, and 4,4'-dihydroxybenzophenone, **7e**, furnished the alkene **10** (49%), but no pinacol rearrangement product was observed (Scheme 3). Once again, however, the chlorine atom was replaced by a hydrogen atom, and the identity of **10** was confirmed by direct coupling of **7e** with acetylferrocene, **11**; in this latter case, **10** was obtained in 90% yield. The corresponding reaction between propionylferrocene, **12**, and ketones **7b** or **7f**, yielded classical alkenes **4** or **13** in yields of 30% or 33%, respectively (Scheme 4). The rearrangement products **14** or **15** were also formed as contaminating by-products, and the X-ray crystal structure of 3-ferrocenyl-3-(4-hydroxyphenyl)-hexan-4-one, **14**, is shown as Figure 3.

**Scheme 5:**
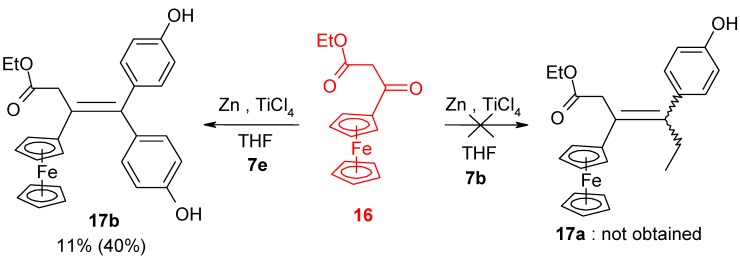
McMurry reaction between ketoester **16** and mono (**7b**) and diaryl (**7e**) ketones.

Having shown that alcohols **5a**–**c** could be obtained in low yields from the chlorinated precursor **6**, we sought a more rational route to these compounds. To improve the yield of **5b**, we envisioned a McMurry cross-coupling reaction between 4-hydroxypropiophenone, **7b**, and ethyl 3-ferrocenyl-3-oxo-propanoate, **16** (Scheme 5), with the aim of reducing the ester **17a** to the required alcohol **5b**. However, McMurry coupling failed to give the desired ester **17a**, the only material isolated being the homocoupling product, *i.e.*, diethylstilbestrol (DES), together with recovered unreacted ketoester **16**.

**Figure 4:**
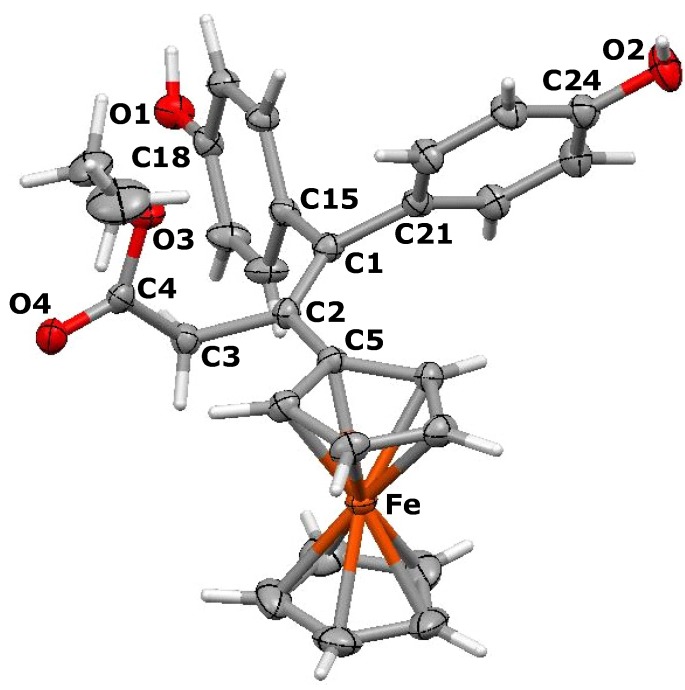
Molecular structure of ethyl 3-ene-3-ferrocenyl-4,4-bis-(4-hydroxyphenyl)-butanoate, **17b**. Thermal ellipsoids are shown at 25%. Selected distances (Å): C(1)-C(2) 1.3609(17), C(1)-C(15) 1.5048(16), C(1)-C(21) 1.5021(17), C(2)-C(5) 1.4852(16), C(2)-C(3) 1.5273(17), C(3)-C(4) 1.5246(18), C(4)-O(3) 1.3360(16), C(4)-O(4) 1.2255(16), C(18)-O(1) 1.3853(15), C(24)-O(2) 1.3771(16), Fe-centroid(C_5_H_4_) 1.662, Fe-centroid(C_5_H_5_) 1.667.

In contrast, the reaction of the ketoester **16** and 4,4'-dihydroxypropiophenone, **7e**, led to the alkene **17b**, but in only 11% yield, and remaining ketoester was recovered. However, by using two equivalents of 4,4'-dihydroxybenzophenone, the cross-coupling yield could be increased to 40%, allowing the obtention of X-ray quality crystals of **17b**, whose structure appears as Figure 4.

The C_5_H_4_ ring of the ferrocenyl substituent is twisted through 27° out of the plane of the double bond, whereas the *Z* and *E* phenolic rings are rotated through 60.4° and 64.3°, respectively, in opposite directions, making a dihedral angle between them of 89.3°. The cyclopentadienyl rings in the ferrocenyl moiety are parallel and are separated by 3.329 Å. The results of all the coupling reactions are summarized in Table 1.

**Table 1:** Collected results from the McMurry coupling reactions.

Entry	Ketone 1	Ketone 2	Product (Yield)	Comments
1	FcC(=O)CH_2_Cl	*p*-HO-C_6_H_4_-C(=O)Et	**9** (29%)	Rearrangement with exchange of Cl → H
2	FcC(=O)CH_2_Cl	(*p*-HO-C_6_H_4_)_2_C=O	**10** (49%)	Alkene with exchange Cl → H
3	FcC(=O)Me	(*p*-HO-C_6_H_4_)_2_C=O	**10** (90%)	Alkene
4	FcC(=O)(CH_2_)_2_Cl	*p*-HO-C_6_H_4_-C(=O)Me	**5a** (10%)	Alkene with exchange Cl → OH
5	FcC(=O)(CH_2_)_2_Cl	*p*-HO-C_6_H_4_-C(=O)Et	**5b** (16%)	Alkene with exchange Cl → OH
6	FcC(=O)(CH_2_)_2_Cl	*p*-HO-C_6_H_4_-C(=O)Pr	**5c** (15%)	Alkene with exchange Cl → OH
7	FcC(=O)(CH_2_)_2_Cl	*p*-HO-C_6_H_4_-C(=O)H	-----	Complex inseparable mixture
8	FcC(=O)(CH_2_)_2_Cl	(*p*-HO-C_6_H_4_)_2_C=O	**2** (47%)	Alkene, no exchange, published work [26]
9	FcC(=O)Et	*p*-HO-C_6_H_4_-C(=O)Et	**4** (30%)	Alkene, and also isolation of a rearrangement product, **14**
10	FcC(=O)Et	*p*-MeO-C_6_H_4_-C(=O)Et	**13** (33%)	Alkene
11	FcC(=O)CH_2_CO_2_Et	*p*-HO-C_6_H_4_-C(=O)Et	-----	Homocoupling (DES)
12	FcC(=O)CH_2_CO_2_Et	(*p*-HO-C_6_H_4_)_2_C=O	**17b** (11%)	Alkene
13	FcC(=O)CH_2_CO_2_Et	(*p*-HO-C_6_H_4_)_2_C=O	**17b** (40%)	Alkene when using 2 eq of (*p*-HOC_6_H_4_)_2_C=O

### 2.2. Mechanistic Considerations

The failure of McMurry reactions of chloroalkyl-ferrocenyl ketones to yield chlorinated products raises interesting questions. In the case of chloroacetylferrocene, the chlorine was replaced by hydrogen (entries 1 and 2 of Table 1), whereas reactions of chloropropionylferrocene with mono-aryl ketones led to hydroxyethyl derivatives **5a**–**c** (entries 4–6).

**Scheme 6:**
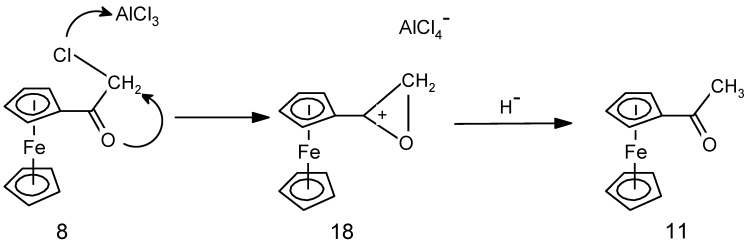
Proposed mechanism for the Lewis acid promoted conversion of a chloromethyl substituent into a methyl group.

We consider first the case of chloroacetylferrocene, **8**, originally prepared by Schlögl and Egger [31] by Friedel-Crafts acylation of ferrocene in the presence of AlCl_3_. In particular, we note their report that, when a solution of **8** in CS_2_ or CH_2_Cl_2_ was stirred with AlCl_3_ for two hours at room temperature, it suffered dechlorination to form acetylferrocene, **11**, whose identity was confirmed by independent synthesis. Apparently, abstraction of chloride by the Lewis acid to form AlCl_4_^−^ leads initially to formation of the corresponding primary carbocation that is presumably stabilized as the strained three-membered cyclic ferrocenyl-stabilized cation **18** (Scheme 6). Reduction to form the methyl group in **11** requires formal addition of a hydride, and such a process is well-known to proceed under Friedel-Crafts conditions whereby abstraction of hydride can occur-even from an alkane! [33]. Now, assuming that a Lewis acidic titanium chloride can behave analogously by abstracting a chloride from **8**, there is a straightforward explanation for the conversion of a chloroacetyl substituent into the corresponding acetyl derivative, as in **10** (entry 2).

**Scheme 7:**
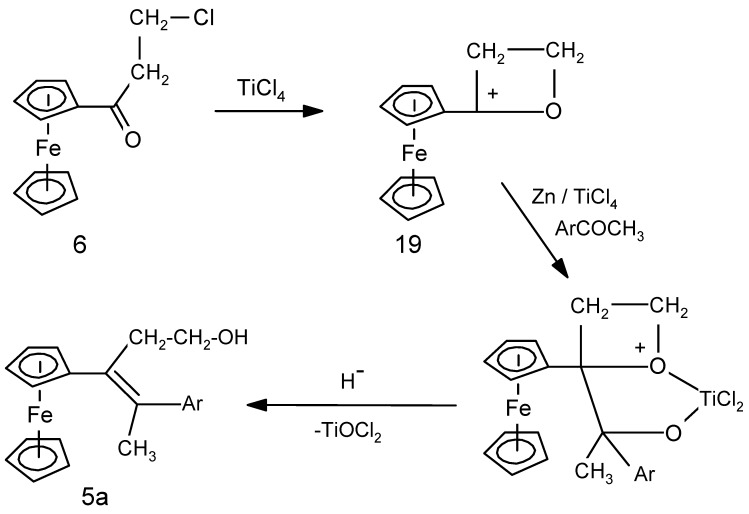
Proposed mechanism for the Lewis acid promoted conversion of a chloroethyl substituent into a hydroxyethyl group.

Turning now to the generation of the hydroxyethyl substituents in **5a**–**c**, once again one can envisage abstraction of chloride to form a primary cation that cyclises to the somewhat less strained four-membered ferrocenyl-stabilized cation **19** (Scheme 7). Attachment of TiCl_2_ to the two ketonic oxygens, addition of hydride and elimination of TiOCl_2_ can lead to the observed products. However, the hydride abstraction process is rather inefficient and the observed yields are low. It is now apparent why hydroxyethylation is observed in reactions of chloropropionylferrocene, **6**, with mono-aryl ketones when there is a possible source of hydride by abstraction from the alkyl group, but is not possible in reactions involving a diaryl ketone. This also provides a rationale for the non-formation of a hydroxyethyl derivative from 4-hydroxybenzaldehyde (entry 7 in Table 1).

### 2.3. Biological Results

The IC_50_ values (µM) of most of the new isolated compounds were measured on hormone-independent breast cancer cells MDA-MB-231, and are collected in Table 2. The IC_50_ values of ferrocidiphenol **1** and ferrociphenol are also included for the sake of comparison [10,34].

**Table 2:** IC_50_ values (µM). a: value from reference [10], b: value from reference [26], c: value from reference [34], d: value from reference [35]

Compound	IC_50_ (μM)	Compound	IC_50_ (μM)
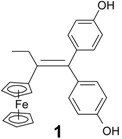	0.60 ± 0.06 ^a^	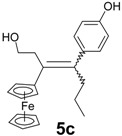	0.36 ± 0.04
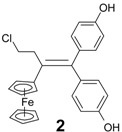	1.00 ± 0.01 ^b^	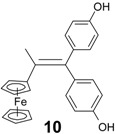	1.09 ± 0.22
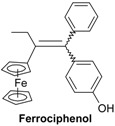	1.13 ± 0.13 ^c^	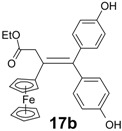	1.16 ± 0.10
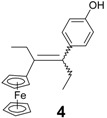	1.14 ± 0.05	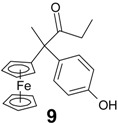	3.13 ± 0.07
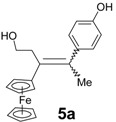	0.14 ± 0.01	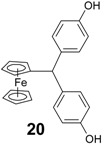	3.5 ± 0.02 ^d^
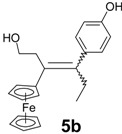	0.28 ± 0.01		

It is noteworthy that compounds **5a**–**c**, which contain the hydroxyethyl group, are more active than the related ferrocidiphenol, **1**, with IC_50_ values for **5a**, **5b** and **5c** of 0.14, 0.28 and 0.36 µM, respectively. Indeed, compound **5a** (IC_50_ = 0.14 μM) is four times more potent than **1** (IC_50_ = 0.60 μM [10]). We have shown previously that compounds of the ferrociphenol type have a higher activity when they possess two para phenol groups (or two biologically hydrolysable ester groups [36]) as compared to compounds bearing only one phenol group [34,37,38]. Hence, as listed in Table 2, the ferrocidiphenol, **1**, with two phenol groups, is more active than its monophenol analogue, Fc(Et)C=CPh(C_6_H_4_OH), which shows an IC_50_ value of 1.13 µM [34]. It appears, therefore, that the hydroxyethyl group imparts a stronger antiproliferative effect than a phenol group. Moreover, it appears that the length of the alkyl chain in the series **5a**–**c** (methyl, ethyl and propyl) plays a significant role; even though the differences are not very large, it seems that the smaller the substituent the more active is the compound. The important role of the hydroxyethyl group is confirmed by the lower activity of compound **4** which lacks the hydroxyl function, whereby its IC_50_ value (1.14 μM) is four times less active than **5b** (IC_50_ = 0.28 μM), and at the same level than the ferrociphenol with a single phenolic substituent. We note that replacement of the ethyl group of the ferrocidiphenol, **1**, by a methyl group in compound **10** led to a decrease in activity. One might have anticipated that the smaller alkyl substituent would have led to somewhat enhanced activity, but the subtle change in electronic effects may have somewhat disfavored formation of the expected quinone methide metabolite [19].

Finally, we note that the pinacolone **9** is not very active, with an IC_50_ value of only 3.13 µM. This again supports the idea that the [ferrocenyl-double bond-phenol] motif is crucial for antiproliferative activity in this series of compounds. Compound **9** has approximately the same IC_50_ as ferrocenyl-di(4-hydroxyphenyl)methane, **20**, (3.5 µM) [35], which also possesses a saturated central carbon impairing electronic circulation.

## 3. Experimental Section

### 3.1. General Information

All reactions and manipulations were carried out under an argon atmosphere using standard Schlenk techniques. THF was distilled over sodium/benzophenone prior to use. Thin layer chromatography was performed on silica gel 60 GF_254_. The semi-preparative HPLC separations were performed on a Shimadzu instrument with a Nucleodur C18 column (length of 25 cm, diameter of 3.2 cm, and particle size of 10 µm) using acetonitrile/water as eluent. IR spectra were obtained on a FT/IR-4100 JASCO 180 spectrometer. ^1^H and ^13^C-NMR spectra were acquired on a Bruker 300 MHz spectrometer, while 2D NMR spectra were obtained on a Bruker 400 MHz instrument. Mass spectrometry was carried out at the “Service de Spectrométrie de Masse” at ENSCP, Paris. High resolution mass spectra (HRMS) and RX structures were acquired in the “Institut Parisien de Chimie Moléculaire (IPCM-UMR 8232)” at the “Université Pierre et Marie Curie”, Paris. Microanalyses were performed by the “Service de Microanalyse ICSN” at Gif sur Yvette, France. Determination of the cytotoxicity of the complexes was performed at IMAGIF (ICSN, Gif sur Yvette, France).

### 3.2. Chemistry

#### General Procedure for McMurry Coupling Reactions

Zinc powder was suspended in dry THF at room temperature in a Schlenk tube under argon, and titanium tetrachloride was added slowly via a syringe while stirring. The reaction mixture was heated at reflux for 2 h, after which time a THF solution containing the two ketones was added, and the mixture was heated at reflux for a further two hours. The reaction mixture was cooled, poured into water, acidified with HCl until the dark color disappeared and extracted with dichloromethane. The organic layer was washed with water, dried over magnesium sulfate, filtered and concentrated under reduced pressure. The crude mixture was chromatographed on silica gel column using a mixture of dichloromethane/acetone 90/10 as an eluent and/or purified by semi-preparative HPLC using a mixture of acetonitrile/water as an eluent.

*3-Ferrocenyl-4-(4-hydroxyphenyl)-hex-3-ene* (**4**) *(and rearrangement product* **14***)*. Zinc powder (1.82 g, 27.9 mmol), titanium tetrachloride (2.04 mL, 18.6 mmol), propionylferrocene (1.13 g, 4.65 mmol), *p*-hydroxypropiophenone (0.70 g, 4.65 mmol). After column chromatography followed by preparative HPLC (acetonitrile/water 90/10), and recrystallization from a pentane/diethylether, **4** was obtained as orange crystals (0.50 g, 30% yield) consisting of a mixture of *Z* and *E* isomers (63/37). ^1^H-NMR (300 MHz, CDCl_3_): δ 0.92 (t, *J* = 7.5 Hz, 3H, CH_3_), 1.24 (t, *J* = 7.5 Hz, 3H, CH_3_), 2.28 and 2.41 (q, *J* = 7.5 Hz, 2H, CH_2_), 2.53 and 2.61 (q, *J* =7.5 Hz, 2H, CH_2_), 3.70 and 4.24 (s, 2H, C_5_H_4_), 3.97 and 4.35 (s, 2H, C_5_H_4_), 4.03 and 4.15 (s, 5H, Cp), 4.70 and 4.72 (s, 1H, OH), 6.77 and 6.82 (d, *J* = 8.5 Hz, 2H, C_6_H_4_), 6.90 and 7.00 (d, *J* = 8.5 Hz, 2H, C_6_H_4_). ^13^C-NMR (75.4 MHz, CDCl_3_): δ 13.0 and 13.2 (CH_3_), 15.3 and 16.0 (CH_3_), 26.7 and 28.4 (CH_2_), 28.8 and 29.5 (CH_2_), 67.8 and 67.9 (2CH, C_5_H_4_), 69.0 and 69.1 (2CH, C_5_H_4_), 69.1 and 69.2 (5CH, Cp), 77.4 (C, C_5_H_4_), 115.1 and 115.2 (2CH, C_6_H_4_), 130.1 and 130.5 (2CH, C_6_H_4_), 133.3 (C), 137.1 and 137.2 (C), 138.0 (C), 153.8 and 153.9 (C). IR (KBr, ν cm^−1^): 3418 (OH), 3094, 3062, 2929, 2871 (CH_2_, CH_3_). MS (EI, 70 eV) *m/z*: 360 [M]^+.^, 331 [M-Et]^+^, 295 [M-Cp]^+^, 121 [CpFe]^+^. MS (CI, CH_4_) *m/z*: 361 [M+H]^+^, 360 [M]^+.^, 295. HRMS (ESI, C_22_H_24_FeO: [M]^+.^) calcd: 360.11765, found: 360.11711.

*3-Ferrocenyl-3-(4-hydroxyphenyl)-hexan-4-one* (**14**). A sample of the transposition product **14** was isolated from the chromatography of the crude mixture of the reaction of McMurry giving **4**. HPLC (acetonitrile/water 80/20) then recrystallization from a pentane/diethylether mixture, **14** was obtainedas orange crystals. ^1^H-NMR (300 MHz, CDCl_3_): δ 0.73 (t, *J* = 7.4 Hz, 3H, CH_3_), 0.97 (t, *J* = 7.2 Hz, 3H, CH_3_), 1.95-2.40 (m, 4H, CH_2_), 3.96 (s, 1H, C_5_H_4_), 4.09 (s, 1H, C_5_H_4_), 4.14 (s, 5H, Cp), 4.22 (s, 1H, C_5_H_4_), 4.25 (s, 1H, C_5_H_4_), 4.85 (s, 1H, OH), 6.79 (d, *J* = 6.6 Hz, 2H, C_6_H_4_), 7.14 (d, *J* = 6.6 Hz, 2H, C_6_H_4_). ^13^C-NMR (75.5 MHz, CDCl_3_): δ 9.0 (CH_3_), 10.3 (CH_3_), 31.1 (CH_2_), 32.9 (CH_2_), 59.8 (C), 67.4 (CH, C_5_H_4_), 67.9 (CH, C_5_H_4_), 68.0 (CH, C_5_H_4_), 69.0 (CH, C_5_H_4_), 69.3 (5CH, Cp), 91.3 (C, C_5_H_4_), 114.7 (2CH, C_6_H_4_), 129.6 (2CH + C, C_6_H_4_), 154.1 (C, C_6_H_4_), 211.4 (CO). IR (KBr, ν cm^−1^): 3454 (OH), 2991, 2971, 2935, 2878 (CH_2_, CH_3_), 1684 (CO). MS (ESI+, NH_3_, C_22_H_24_FeO_2_) *m/z*: 376 [M]^+.^, 394 [M+NH_4_]^+^, 399 [M+Na]^+^. MS (ESI-, C_22_H_24_FeO_2_) *m/z*: 375 [M-H]^−^, 751 [2M-H]^−^. Anal. Calcd for C_22_H_24_FeO_2_.(H_2_O)_0.1_: C, 69.89; H, 6.45. Found: C, 69.61; H, 6.36.

*3-Ferrocenyl-4-(4-hydroxyphenyl)-1-hydroxy-pent-3-ene* (**5a**). Zinc powder (2.35 g, 36 mmol), titanium tetrachloride (2.64 mL, 24 mmol), ferrocenyl 2-chloroethyl ketone (1.66 g, 6 mmol), *p*-hydroxyacetophenone (0.82 g, 6 mmol). HPLC (acetonitrile/water 80/20) then recrystallization from a pentane/diethylether mixture, **5a** was obtained as bright yellow crystals (0.21 g, 10% yield) consisting of a mixture of *Z* and *E* isomers (73/27). ^1^H-NMR (300 MHz, acetone-*d*_6_): δ 2.11 (s, 3H, CH_3_), 2.68 and 2.97 (t, *J* = 8.7 Hz, 2H, CH_2_), 3.41–3.55 and 3.74–3.83 (m, 2H, CH_2_O), 3.81 and 4.28 (t, *J* = 1.8 Hz, 2H, C_5_H_4_), 3.98 and 4.43 (t, *J* = 1.8 Hz, 2H, C_5_H_4_), 4.07 and 4.20 (s, 5H, Cp), 6.75 and 6.85 (d, *J* = 8.7 Hz, 2H, C_6_H_4_), 6.88 and 7.06 (d, *J* = 8.7 Hz, 2H, C_6_H_4_), 8.19 and 8.24 (s, 1H, OH). ^13^C-NMR (75.4 MHz, acetone-*d*_6_): δ 23.0 (CH_3_), 38.9 (CH_2_), 62.6 (CH_2_O), 68.1 (2CH, C_5_H_4_), 69.5 (5CH, Cp), 69.7 (2CH, C_5_H_4_), 86.7 (C, C_5_H_4_), 115.9 (2CH, C_6_H_4_), 130.4 (2CH, C_6_H_4_), 145.1 (C), 156.4 (C), 157.0 (C), 161.0 (C). IR (KBr, ν cm^−1^): 3444 (OH), 2959, 2933, 2882 (CH_2_, CH_3_). HRMS (CI, CH_4_, C_21_H_23_FeO_2_: [M+H]^+^) calcd: 363.1048, found: 363.1047. Anal. Calcd for C_21_H_22_FeO_2_.H_2_O: C, 66.32; H, 6.36. Found: C, 66.63; H, 6.35.

*3-Ferrocenyl-4-(4-hydroxyphenyl)-1-hydroxy-hex-3-ene* (**5b**). Zinc powder (0.79 g, 12 mmol), titanium tetrachloride (1.51 mL, 8 mmol), ferrocenyl 2-chloroethyl ketone (0.55 g, 2 mmol), *p*-hydroxypropiophenone (0.30 g, 2 mmol). HPLC (acetonitrile/water 80/20) then recrystallization from a pentane/diethylether mixture, **5b** was obtained as bright yellow crystals (0.12 g, 16% yield) consisting of a mixture of *Z* and *E* isomers (86/14). ^1^H-NMR (300 MHz, acetone-*d*_6_): δ 0.93 and 0.94 (t, *J* = 7.4 Hz, 2H, CH_3_), 2.50 and 2.55 (q, *J* = 7.4 Hz, CH_2_), 2.68 and 2.97 (t, *J* = 7.9 Hz, 2H, CH_2_), 3.39–3.58 and 3.77–3.86 (m, 2H, CH_2_OH), 3.80 and 4.30 (t, *J* = 1.9 Hz, 2H, C_5_H_4_), 4.00 and 4.43 (t, *J* = 1.9 Hz, 2H, C_5_H_4_), 4.08 and 4.23 (s, 5H, Cp), 6.82 and 6.88 (d, *J* = 8.6 Hz, 2H, C_6_H_4_), 6.90 and 7.04 (d, *J* = 8.6 Hz, 2H, C_6_H_4_), 8.23 and 8.26 (s, 1H, OH). ^13^C-NMR (75.4 MHz, acetone-d_6_): δ 13.9 and 14.2 (CH_3_), 29.8 and 30.6 (CH_2_), 40.8 (CH_2_), 63.4 and 64.2 (CH_2_O), 69.0 and 69.3 (2CH, C_5_H_4_), 70.4 and 70.6 (5CH, Cp), 70.5 and 70.6 (2CH, C_5_H_4_), 90.1 and 91.1 (C, C_5_H_4_), 116.5 and 116.7 (2CH, C_6_H_4_), 131.3 and 131.7 (2CH, C_6_H_4_), 136.3 and 136.7 (C), 141.7 (C), 143.6 (C), 157.4 and 157.5 (C). MS (EI, 70 eV) *m/z*: 376 [M]^+.^. Anal. Calcd. for C_22_H_24_O_2_Fe: C, 70.23; H, 6.43. Found: C, 70.48; H, 6.69. A second recrystallization gave pure major isomer which was identified as the *Z* isomer by 2D NMR experiments.

*3-Ferrocenyl-4-(4-hydroxyphenyl)-1-hydroxy-hept-3-ene* (**5c**). Zinc powder (3.14 g, 48 mmol), titanium tetrachloride (3.52 mL, 32 mmol), ferrocenyl 2-chloroethyl ketone (2.21 g, 8 mmol), *p*-hydroxybutyrophenone (1.31 g, 8 mmol). HPLC (acetonitrile/water 80/20) then recrystallization from a pentane/diethylether mixture, **5c** was obtained as bright yellow crystals (0.45 g, 15% yield) consisting of a mixture of *Z* and *E* isomers (62/38). ^1^H-NMR (300 MHz, acetone-d_6_): δ 0.89 (t, *J* = 7.4 Hz, 3H, CH_3_), 1.29–1.33 (m, 2H, CH_2_), 2.40 and 2.45 (t, *J*= 7.4, 2H, CH_2_), 2.65 and 2.93 (t, *J* = 7.4, 2H, CH_2_), 3.36–3.53 and 3.75–3.82 (m, 2H, CH_2_O), 3.76 and 4.26 (t, *J* = 1.8 Hz, 2H, C_5_H_4_), 3.96 and 4.36 (t, *J* = 1.8 Hz, 2H, C_5_H_4_), 4.04 and 4.19 (s, 5H, Cp), 6.78 and 6.84 (d, *J* = 8.7 Hz, 2H, C_6_H_4_), 6.86 and 7.00 (d, *J* = 8.7 Hz, 2H, C_6_H_4_), 8.20 and 8.22 (s, 1H, OH). ^13^C-NMR (75.4 MHz, acetone-d_6_): δ 15.1 and 15.2 (CH_3_), 22.8 and 23.0 (CH_2_), 39.1 and 40.1 (CH_2_), 39.6 and 40.9 (CH_2_), 63.4 and 64.2 (CH_2_O), 69.0 and 69.2 (2CH, C_5_H_4_), 70.3 and 70.4 (5CH, Cp), 70.5 and 70.6 (2CH, C_5_H_4_), 89.6 and 90.3 (C, C_5_H_4_), 116.6 and 116.7 (2CH, C_6_H_4_), 131.2 and 131.6 (2CH, C_6_H_4_), 137.2 (C), 140.3 (C), 142.4 (C), 157.5 (C). IR (KBr, ν cm^−1^): 3418 (OH), 2959, 2870 (CH_2_, CH_3_). HRMS (CI, CH_4_, C_23_H_27_FeO_2_: [M+H]^+^) calcd: 391.1361, found: 391.1359. Anal. Calcd for C_23_H_26_FeO_2_: C, 70.77; H, 6.71. Found: C, 70.49; H, 6.75.

*2-Ferrocenyl-2-(4-hydroxyphenyl)-pentan-3-one* (**9**). Zinc powder (2.35 g, 36 mmol), titanium tetrachloride (2.64 mL, 24 mmol), chloroacetylferrocene (1.58 g, 6.0 mmol), *p*-hydroxypropiophenone (0.90 g, 6.0 mmol). After purification by HPLC (acetonitrile/water 80/20), and recrystallization from pentane/diethylether mixture, **9** was obtained as orange crystals (0.62 g, 29% yield). ^1^H-NMR (300 MHz, acetone-d_6_): δ 0.97 (t, *J* = 7.5 Hz, 3H, CH_3_), 1.77 (s, 3H, CH_3_), 2.32-2.38 (m, 2H, CH_2_), 4.08 (s, 1H, C_5_H_4_), 4.15 (s, 5H, Cp), 4.27 (s, 3H, C_5_H_4_), 6.79 (d, *J* = 8.4 Hz, 2H, C_6_H_4_), 6.99 (d, *J* = 8.4 Hz, 2H, C_6_H_4_), 8.30 (s, 1H, OH). ^13^C-NMR (75.4 MHz, acetone-*d*_6_): δ 9.4 (CH_3_), 26.4 (CH_3_), 33.5 (CH_2_), 56.5 (C), 68.2 (CH, C_5_H_4_), 68.3 (CH, C_5_H_4_), 68.9 (CH, C_5_H_4_), 69.0 (CH, C_5_H_4_), 69.7 (5CH, Cp), 92.5 (C, C_5_H_4_), 115.8 (2CH, C_6_H_4_), 129.0 (2CH, C_6_H_4_), 137.1 (C, C_6_H_4_), 156.9 (C, C_6_H_4_), 211.4 (CO). IR (KBr, ν cm^−1^): 3342 (OH), 3097, 2986, 2937 (CH_2_, CH_3_), 1687 (CO). MS (CI, CH_4_) *m/z*: 363 [M+H]^+^, 362 [M]^+.^, 391 [M+C_2_H_5_]^+^, 305, 269, 186. Anal. Calcd for C_21_H_22_FeO_2_: C, 69.62; H, 6.12. Found: C, 69.37; H, 6.14.

*2-Ferrocenyl-1,1-bis-(4-hydroxyphenyl)-prop-1-ene* (**10**). Zinc powder (3.56 g, 54.5 mmol), titanium tetrachloride (3.2 mL, 29 mmol), acetylferrocene (2 g, 8.9 mmol), 4,4'-dihydroxybenzophenone (1.9 g, 8.9 mmol). After chromatography, recrystallization from a pentane/diethylether mixture, **10** was obtained as bright yellow crystals (3.26 g, 90% yield). m.p. 94–95 °C; ^1^H-NMR (300 MHz, acetone-*d*_6_): δ 2.20 (s, 3H, CH_3_), 3.97 (t, *J* = 1.9 Hz, 2H, C_5_H_4_), 4.09 (t, *J* = 1.9 Hz, 2H, C_5_H_4_), 4.17 (s, 5 H, Cp), 6.72 (d, *J* = 8.5 Hz, 2H, C_6_H_4_), 6.84 (d, *J* = 8.5 Hz, 2H, C_6_H_4_), 6.85 (d, *J* = 8.5 Hz, 2H, C_6_H_4_), 7.06 (d, *J* = 8.5 Hz, 2H, C_6_H_4_), 8.22 (s, 1H, OH), 8.29 (s, 1H, OH). ^13^C-NMR (75.4 MHz, acetone-*d*_6_): δ 22.2 (CH_3_), 68.5 (2CH, C_5_H_4_), 69.7 (5CH, Cp), 69.9 (2CH, C_5_H_4_), 89.0 (C, C_5_H_4_), 115.6 (2 × 2CH, C_6_H_4_), 129.8 (C), 131.8 (2CH, C_6_H_4_), 132.2 (2CH, C_6_H_4_), 136.9 (C), 137.0 (C), 138.8 (C), 156.7 (2C). IR (KBr, ν cm^−1^): 3378 (OH). MS (EI, 70 eV) *m/z*: 410 [M]^+.^, 395 [M-Me]^+^, 345 [M-Cp]^+^, 121 [CpFe]^+^. HRMS (FAB, C_25_H_22_FeO_2_: [M]^+^) calcd: 410.0969, found: 410.0986. Anal. Calcd for C_25_H_22_FeO_2_.H_2_O: C, 70.10; H, 5.64. Found: C, 70.45; H, 5.55.

*3-Ferrocenyl-4-(4-methoxyphenyl)-hex-3-ene* (**13**). Zinc powder (1.82 g, 27.9 mmol), titanium tetrachloride (2.04 mL, 18.6 mmol), ferrocenyl ethyl ketone (1.13 g, 4.65 mmol), *p*-methoxypropiophenone (0.76 g, 4.6 mmol). Chromatography then HPLC (acetonitrile/water 70/30). Recrystallization from EtOH gave **13** as orange crystals (0.57 g, 33% yield) consisting of a mixture of *Z* and *E* isomers (71/29). ^1^H-NMR (300 MHz, CDCl_3_): δ 0.94 (t, *J* = 7.4 Hz, 3H, CH_3_), 1.27 (t, *J* = 7.4 Hz, 3H, CH_3_), 2.30 and 2.43 (q, *J* = 7.4 Hz, 2H, CH_2_), 2.56 and 2.63 (q, *J* = 7.4 Hz, 2H, CH_2_), 3.72 and 4.26 (s, 2H, C_5_H_4_), 3.83 and 3.85 (s, 3H, OCH_3_), 3.99 and 4.37 (s, 2H, C_5_H_4_), 4.05 and 4.16 (s, 5H, Cp), 6.86 and 6.91 (d, *J* = 8.6 Hz, 2H, C_6_H_4_), 6.97 and 7.07 (d, *J* = 8.6 Hz, 2H, C_6_H_4_). ^13^C-NMR (75.4 MHz, CDCl_3_): δ 12.8 and 13.0 (CH_3_), 15.2 and 15.9 (CH_3_), 26.5 and 28.3 (CH_2_), 28.7 and 29.3 (CH_2_), 55.1 (OCH_3_), 67.5 and 67.7 (2CH, C_5_H_4_), 68.7 and 68.8 (2CH, C_5_H_4_), 68.9 and 69.0 (5CH, Cp), 87.5 and 87.7 (C, C_5_H_4_), 113.4 and 113.5 (2CH, C_6_H_4_), 129.7 and 130.1 (2CH, C_6_H_4_), 133.0 and 133.3 (C), 136.7 and 136.9 (C), 137.9 and 139.8 (C), 157.7 and 157.9 (C). IR (KBr, ν cm^−1^): 3094, 3062, 2929, 2871 (CH_2_, CH_3_). MS (EI, 70 eV) *m/z*: 374 [M]^+.^, 345, 309 [M-Cp]^+^, 293, 121 [CpFe]^+^. Anal. Calcd for C_23_H_26_FeO: C, 73.80; H, 7.00. Found: C, 73.44; H, 6.94.

*Ethyl 3-ene-3-ferrocenyl-4,4-bis-(4-hydroxyphenyl)-butanoate* (**17b**). The general procedure has been followed. Zinc powder (7.84 g, 120 mmol), titanium tetrachloride (8.8 mL, 80 mmol), ethyl 3-ferrocenyl-3-oxo-propanoate (6 g, 20 mmol), 4,4'-dihydroxybenzophenone (8.6 g, 40 mmol). After chromatography, recrystallization from EtOH/water, **17b** was obtained as orange crystals (3.84 g, 40% yield). ^1^H-NMR (acetone-*d*_6_): δ 1.23 (t, *J* = 7.2 Hz, 3H, CH_3_), 3.65 (s, 2H, CH_2_), 3.93 (t, *J* = 2.0 Hz, 2H, C_5_H_4_), 4.09 (q, *J* = 7.2 Hz, 2H, CH_2_O), 4.11 (t, *J* = 2.0 Hz, 2H, C_5_H_4_), 4.17 (s, 5H, Cp), 6.76 (d, *J* = 8.7 Hz, 2H, C_6_H_4_), 6.83 (d, *J* = 8.7 Hz, 2H, C_6_H_4_), 6.97 (d, *J* = 8.7 Hz, 2H, C_6_H_4_), 7.10 (d, *J* = 8.7 Hz, 2H, C_6_H_4_), 8.31 (s, 1H, OH), 8.33 (s, 1H, OH). ^13^C-NMR (acetone-*d*_6_): δ 15.3 (CH_3_), 44.1 (CH_2_), 61.5 (CH_2_O), 69.5 (2CH, C_5_H_4_), 70.6 (5CH, Cp), 70.7 (2CH, C_5_H_4_), 88.8 (C, C_5_H_4_), 116.5 (2CH, C_6_H_4_), 116.6 (2CH, C_6_H_4_), 128.9 (C), 131.8 (2CH, C_6_H_4_), 132.4 (2CH, C_6_H_4_), 137.2 (C), 137.3 (C), 142.8 (C), 157.7 (2C), 173.2 (CO). IR (KBr, ν cm^−1^): 3361 (OH), 2933, 2986, 3035 (CH_2_, CH_3_), 1682 (CO). MS (CI, NH_3_) *m/z*: 483 [M+H]^+^, 500 [M+NH_4_]^+^. HRMS (CI, NH_3_, C_28_H_27_FeO_4_: [M+H]^+^) calcd: 483.1259, found: 483.1265. Anal. Calcd for C_28_H_26_FeO_4_: C, 69.72; H, 5.43. Found: C, 69.63; H, 5.45.

### 3.3. X-ray Crystallography

Suitable crystals of **5b**, **9**, **14** and **17b** were obtained from acetonitrile or acetone:hexane (for **5b**). Crystallographic data are given in Table S1 in the Supporting Information. These have also been deposited at the Cambridge Crystallographic Data Centre: **5b** (1007086), **9** (1007087) and **17b** (1007088). Molecule **14** exhibited a minor crystallographic disorder giving rise to an apparent mirror plane so, although the structure is unambiguously established, we prefer not to quote precise bond lengths and angles. Unit cell data are listed in reference [39].

## 4. Conclusions

It has been shown here that the ferrocenyl-diaryl-ethylene series is not the only one active against hormone-independent breast cancer cells. The second *p*-hydroxyphenyl group can be replaced by an alkyl group, but in this case the presence of a hydroxyethyl group is necessary for good activity. In fact, the alkyl group seems not to play an essential role except for a decrease in activity as the chain lengthens. As a result of these factors, 3-ferrocenyl-4-(4-hydroxyphenyl)-1-hydroxy-pent-3-ene, **5a**, bearing a methyl group adjacent to the phenol substituent, has the best value of the series (IC_50_ = 0.14 µM). Unfortunately, at present the yields are low for the preparation of these promising hydroxylated species (10%–16% for the McMurry coupling), and need to be improved.

Even though one starts from relatively inexpensive, commercially available ketones and easily synthesized chloropropionyl-ferrocene, **6**, (Friedel-Crafts), and the McMurry second step led to the cross-coupled product possessing the double bond and the hydroxyethyl substituent in a one-pot process, nevertheless, final separation of the desired product is difficult. Modified reaction conditions, together with an enhanced understanding of the mechanism (such as the importance of hydride transfer) may lead to improved yields. It may be worthwhile to attempt the McMurry reaction starting directly from the keto-alcohol, but pinacolone by-products would of course diminish the overall yields, and alternative routes need to be developed. Other new compounds bearing the [ferrocenyl-(hydroxyethyl)-ene-phenol] motif are under active investigation with the aim of elucidating the role of the hydroxyethyl group in promoting cytotoxic activity.

## Supplementary Materials

Supplementary materials can be accessed at:  http://www.mdpi.com/1420-3049/19/7/10350/s1.
