# Supplementary material for: Atypical McMurry Cross-Coupling Reactions Leading to a New Series of Potent Antiproliferative Compounds Bearing the Key [Ferrocenyl-Ene-Phenol] Motif
Source: Molecules. 2014 Jul 17;19(7):10350–69. doi: 10.3390/molecules190710350 (PMC6271948; doi:10.3390/molecules190710350)
Supplement: Supplementary file 1 [file molecules-19-10350-s001.pdf]

Supplementary Information

Figure S1. NMR COSY spectrum of **5b**.

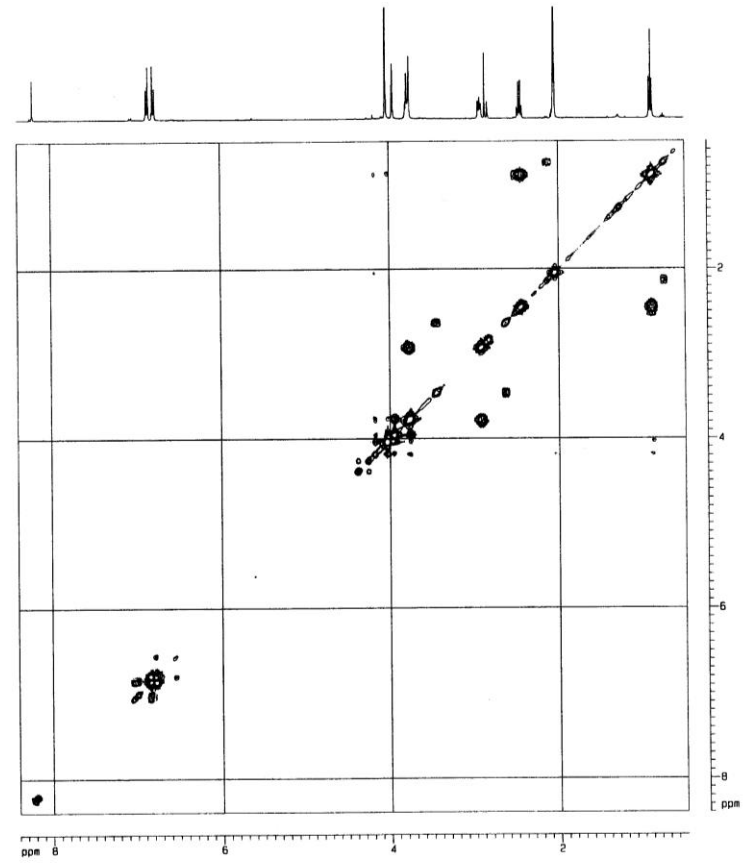

Figure S2. NMR NOESY spectrum of **5b**.

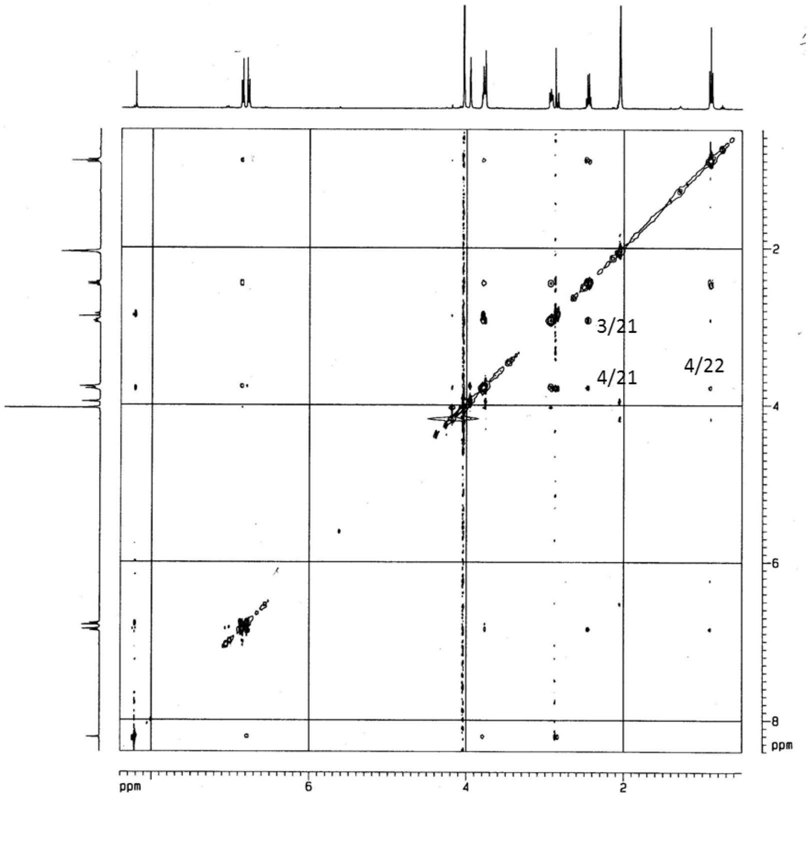

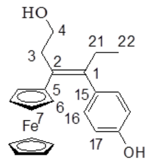

5b

Table S1. Crystallographic Data for **5b**, **9** and **17b**.

Compound	5b	9	17b
empirical formula	$C_{44}H_{48}O_4Fe_2$ H_2O	$C_{21}H_{22}O_2Fe$	$C_{28}H_{26}O_4Fe$
fw	770.56	362.25	482.36
crystal system	triclinic	monoclinic	triclinic
space group	$P\bar{1}$ (#2)	$P2_1/n$ (#14)	$P\bar{1}$ (#2)
a, Å	10.0902(10)	10.3239(11)	9.4648(9)
b, Å	12.5029(10)	11.5983 (11)	11.2957(13)
c, Å	16.4308(10)	15.2103(15)	11.9089(16)
α, deg	101.317(10)	90	90.915(9)
β, deg	104.243(10)	91.887(10)	92.106(7)
γ, deg	95.653(2)	90	108.490(7)
volume ($Å^3$)	1946.4(3)	1820.3(3)	1206.1(2)
Z	2	4	2
density (calc; g cm^{-3})	1.311	1.322	1.328
temp, (K)	295(2)	200(2)	200(2)
abs coeff (mm^{-1})	0.789	0.837	0.656
F(000)	808	760	504
θ range (deg)	1.31 to 24.97	2.21 to 32.00	1.90 to 30.00
index ranges	$-11 \le h \le 11$	$-15 \le h \le 15$	$-13 \le h \le 13$
	$-14 \le k \le 14$	$-17 \le k \le 17$	$-15 \le k \le 15$
	$-19 \le l \le 19$	$-22 \le l \le 22$	$-16 \le l \le 16$
reflns measured	7328	22060	45650
reflns used (R_{int})	6812	6298	7004
restraints	0	0	0
parameters	471	227	299
final R values I > 2σ(I)]: R1, wR2	0.0504, 0.0691	0.0480, 0.1136	0.0303, 0.0770
R values (all data): R1, wR2	0.0785, 0.0784	0.0962, 0.1397	0.0401, 0.0850
goodness-of-fit on F^2	0.9938	0.8662	0.9590
